# Spatial Distribution and Risk Factors of Highly Pathogenic Avian Influenza (HPAI) H5N1 in China

**DOI:** 10.1371/journal.ppat.1001308

**Published:** 2011-03-03

**Authors:** Vincent Martin, Dirk U. Pfeiffer, Xiaoyan Zhou, Xiangming Xiao, Diann J. Prosser, Fusheng Guo, Marius Gilbert

**Affiliations:** 1 Emergency Centre for the Control of Transboundary Animal Diseases, Food and Agriculture Organization of the United Nations (FAO), Beijing, China; 2 Veterinary Epidemiology & Public Health Group, Department of Veterinary Clinical Sciences, The Royal Veterinary College, University of London, London, United Kingdom; 3 Department of Botany and Microbiology, Center for Spatial Analysis, University of Oklahoma, Norman, Oklahoma, United States of America; 4 USGS Patuxent Wildlife Research Center, Beltsville, Maryland, United States of America; 5 University of Maryland, College Park, Maryland, United States of America; 6 Biological Control and Spatial Ecology, Université Libre de Bruxelles, Brussels, Belgium; 7 Fonds National de la Recherche Scientifique, Brussels, Belgium; Imperial College London, United Kingdom

## Abstract

Highly pathogenic avian influenza (HPAI) H5N1 was first encountered in 1996 in Guangdong province (China) and started spreading throughout Asia and the western Palearctic in 2004–2006. Compared to several other countries where the HPAI H5N1 distribution has been studied in some detail, little is known about the environmental correlates of the HPAI H5N1 distribution in China. HPAI H5N1 clinical disease outbreaks, and HPAI virus (HPAIV) H5N1 isolated from active risk-based surveillance sampling of domestic poultry (referred to as HPAIV H5N1 surveillance positives in this manuscript) were modeled separately using seven risk variables: chicken, domestic waterfowl population density, proportion of land covered by rice or surface water, cropping intensity, elevation, and human population density. We used bootstrapped logistic regression and boosted regression trees (BRT) with cross-validation to identify the weight of each variable, to assess the predictive power of the models, and to map the distribution of HPAI H5N1 risk. HPAI H5N1 clinical disease outbreak occurrence in domestic poultry was mainly associated with chicken density, human population density, and elevation. In contrast, HPAIV H5N1 infection identified by risk-based surveillance was associated with domestic waterfowl density, human population density, and the proportion of land covered by surface water. Both models had a high explanatory power (mean AUC ranging from 0.864 to 0.967). The map of HPAIV H5N1 risk distribution based on active surveillance data emphasized areas south of the Yangtze River, while the distribution of reported outbreak risk extended further North, where the density of poultry and humans is higher. We quantified the statistical association between HPAI H5N1 outbreak, HPAIV distribution and post-vaccination levels of seropositivity (percentage of effective post-vaccination seroconversion in vaccinated birds) and found that provinces with either outbreaks or HPAIV H5N1 surveillance positives in 2007–2009 appeared to have had lower antibody response to vaccination. The distribution of HPAI H5N1 risk in China appears more limited geographically than previously assessed, offering prospects for better targeted surveillance and control interventions.

## Author Summary

The geographical distribution of highly pathogenic avian influenza (HPAI) H5N1 and agro-ecological risk factors have been studied in a number of countries in Southeast Asia. However, little is know of its distribution in China where HPAI H5N1 first emerged in 1996, evolved, and spread throughout Asia and the western Palearctic in 2004–2006. This study analyzes separately the distribution, in domestic poultry, of HPAI virus (HPAIV) H5N1 isolated from active risk-based surveillance sampling and HPAI H5N1 clinical disease outbreaks. These data are analyzed in relation to the distribution of chicken and domestic waterfowl population density, proportion of land covered by rice or surface water, cropping intensity, elevation, and human population density. HPAI H5N1 viruses identified by risk-based surveillance are found to be associated with domestic waterfowl density, human population density, and the proportion of land covered by surface water. In contrast, HPAI H5N1 clinical disease outbreak occurrences were mainly associated with chicken density, human population density, and low elevation. These results show that the distribution of HPAI H5N1 risk in China appears more limited geographically than previously assessed, offering prospects for better targeted surveillance and control interventions.

## Introduction

HPAI H5N1 virus infection was first encountered in China in 1996 in the southern part of the country with the discovery of a virus that killed geese in Guangdong province (Goose/GD/96) [1]. In 1997, Hong-Kong experienced the first major outbreak of HPAI H5N1 associated with several human deaths, alerting the international community to the potential threat caused by this new strain of HPAI virus (HPAIV). Between 1999 and 2003, the virus underwent a series of evolutionary changes and multiple genotypes of HPAIV H5N1 detected through routine live bird market surveillance in southern China emerged, indicating that the virus was still active and widely circulating [2]. However, the first major outbreak of HPAI H5N1 in mainland China started in January 2004 in Guangxi autonomous region, in southern China, bordering Vietnam. As the outbreak unfolded, the disease was detected widely throughout the country, causing over 110 outbreaks in 23 provinces since the onset of the epidemic and leading to the culling of more than 35 million poultry to curb the spread of the disease.

To answer the challenge of controlling HPAI H5N1 across such a vast territory characterized by a diversity of agricultural production systems and economic development, China has taken several important steps to confront and control outbreaks and deal with the occurrence of human cases. These steps include measures such as stamping out, movement controls, cleaning and disinfection of infected premises, and the adoption of a nationwide massive vaccination campaign combined with intensive post-vaccination surveillance efforts. Effective vaccines have been developed and disease outbreaks have been responded to in a timely and effective manner, improving China's capacity to contain the disease and drastically reducing the number of outbreaks over the past years, with no outbreak detected in 2010. The year 2005 represented a turning point in the control strategy with the enforcement of a so-called universal vaccination campaign, when vaccination became compulsory for all poultry using the H5N1 Re-1 (A/Goose/Guangdong/1/96-PR8) vaccine strain. In parallel, a large amount of surveillance testing has been conducted both at provincial and national levels with the collection of an average of 4.7 million samples every year during the period 2007–2009 for the detection of silent viral circulation, the possible emergence of new strains and ultimately the identification of potential vaccination failure. Through the national surveillance program for the monitoring of HPAIV H5N1 circulation, the virus has been regularly detected, generating essential information for understanding the infection distribution pattern. More specifically, it has provided evidence that, despite a reduction in reported HPAI H5N1 outbreaks, some parts of the country still offer a favorable breeding ground for influenza viruses to circulate and potentially novel strains to emerge, representing a threat for the generation of new influenza pandemic strains. This particularly applies to the southern part of the country, which has historically been referred to as a hypothetical influenza epicenter [3] where agricultural and cultural practices place man and animals in close proximity. However, very few studies have actually attempted to map the potential distribution of avian influenza disease and infection risk across the diversity of ecological, cultural and production systems present in China. This lack of a sound description of HPAIV H5N1 geographical niches makes it difficult to refine control strategies that rely heavily on vaccination and that would greatly benefit from more targeted risk management. Better understanding of the infection dynamic pattern, the environmental and ecological factors associated with persistence of the disease in various poultry production systems will significantly strengthen efforts to achieve disease control and exclude infection from major poultry production centers, thus optimizing resources allocated to controlling the disease and reducing the risk for human infection.

This study aimed to analyze the interrelationship of HPAI H5N1 in China with its environment, by exploring the association between selected spatial risk factors and two different indicators of HPAIV H5N1 presence, namely reported clinical outbreak occurrence in poultry and detection of sub-clinical HPAIV infection through risk-based surveillance. The study benefits from several improvements over previous work [4]. First, the analysis is not only based on HPAI H5N1 outbreak data which are of limited value in a context of massive vaccination (especially after the implementation of the national vaccination campaign which began towards the end of 2005), but also uses the results of HPAI H5N1 monitoring implemented as part of the national active surveillance program in live bird markets from 2007 to 2009, termed risk-based surveillance in the rest of this manuscript. We also make use of an estimation of vaccine efficacy as measured by the Haemagglutination Inhibition (HI) test of serological samples collected monthly from poultry at province level. Second, we use updated poultry census data that differentiates between chicken, ducks and geese. This distinction is important as shown by previous HPAI H5N1 disease mapping efforts [5], [6], and is probably associated with differences in susceptibility to HPAI H5N1 virus between these species. Third, we used and compared the outputs of bootstrapped logistic regression and boosted regression trees with cross-validation, so that we could robustly estimate the weight of each tested risk factor, the goodness of fit of our predictions, and to allow us to map both the prediction of risk as well as its uncertainty.

## Methods

### Data

Two types of data relating to HPAI H5N1 presence have been used as dependent variables in this study.

First, poultry HPAI H5N1 disease outbreak data were compiled from two main sources: (1) one being the Official Veterinary bulletin published on the website of the Chinese Ministry of Agriculture (MoA; http://www.agri.gov.cn/); and (2) the other source coming from official reports to the World Organisation for Animal Health (OIE) that were compared with MoA's report. Where there was an inconsistency in the outbreak date or location, we obtained accurate data through web research and consultation of local experts. Ninety-five percent of poultry outbreak data had detailed address information which was then geocoded. The remaining 5% for which no accurate location could be obtained were geocoded using the prefecture centroid (administrative level 4).

Second, the Ministry of Agriculture in China routinely coordinates a surveillance program twice a year at the national level and monthly at provincial level in live bird markets consisting of sampling domestic poultry for the detection of HPAIV H5N1. The selection of markets is based on their characteristics with regard to size, trade and hygiene practices which are assumed to increase the likelihood of detecting the virus. All samples collected at provincial level are tested by polymerase chain reaction (PCR). All AI positive samples are sent to the Harbin National Veterinary Research Institute for confirmation, subtyping and virus isolation. The positive HPAIV H5N1 findings are then reported at the central government level and the data are released by the Veterinary Bureau in the MOA through the monthly Official Veterinary Bulletin, from which we extracted data on positive identification of HPAIV H5N1 between January 2007 and September 2009. The surveillance data were geocoded using the market location when the market name was available. For 15% of the data, however, this information was missing and positives were geocoded using the prefecture centroid.

In addition to HPAI H5N1 positives, the monthly proportion of post-vaccination seropositive samples was also extracted from the Monthly Official Veterinary Bulletin at the province level. Post-vaccination monitoring is performed on a regular basis at provincial level to assess the efficacy of the vaccination. Chickens, ducks and geese are sampled 21 days post-immunisation and an effective immune response is defined as a sero-conversion in bird with titres >4Log_2_ when measured by HI test, using homologous antigen, similar to the vaccine strain. Similar to the surveillance results, post-vaccination serological results obtained are collected at national level and published in the Official Veterinary Bulletin.

The spatial distribution of HPAI H5N1 was investigated using a set of 7 explanatory variables which are known to be important risk factors, based on published scientific evidence and expert opinion. First, we considered the abundance of chickens, and domestic waterfowl separately based on previous work that had demonstrated a weak positive association between HPAI H5N1 presence and chicken density [6], [7], but a stronger association with duck density [5]. Second, anthropogenic variables were found to be associated with HPAI H5N1 in a number of studies conducted in countries with very different agro-ecological conditions such as Thailand, Bangladesh, Vietnam, and Romania [6], [8], [7], [9], [10], and we therefore chose to include human population density. Third, several studies also identified land use and cropping variables as significant predictors of HPAI H5N1 presence in Asia. For example, Pfeiffer et al. [7] found HPAI H5N1 to be associated with the proportion of land occupied by aquaculture and by rice paddy fields in Vietnam, and Gilbert et al. [6] found a strong association with rice cropping intensity in Thailand. Similarly, statistically significant effects of access to water, or density of waterways were identified by Biswas et al [11] for Bangladesh and Ward et al. [12] for Romania. Therefore, we decided to include three variables: the proportion of land occupied by water (running water or water bodies), the proportion of land occupied by rice paddy fields, and the cropping intensity (number of crops cultivated in an unit area of cropland over a year). Finally, we included elevation in our analysis since several studies have reported an increased HPAI H5N1 risk in lowland and river delta areas [6], [7], [13]. The risk factor variables and corresponding data sources are presented in Table 1.

**Table 1:** Risk factor variables used in the analysis.

Abbreviation	Description	Transform	Source
ChDnLg	Chickens/km^2^	Log10[x+1]	Robinson et al. [42]
DuGeDnLg	Domestic waterfowls/km^2^	Log10[x+1]	Robinson et al. [42]
HpDnLg	People/km^2^	Log10[x+1]	GRUMP [43]
WaPc	% land occupied by water	-	Jiyuan et al. [44]
RiPc	% land occupied by rice crop	-	Jiyuan et al. [44]
Cint	Average cropping intensity	-	Xiao et al. [45]
DemLg	Mean Elevation	Log10[x+1]	GTOPO30 [46]

The analysis was carried out at a spatial resolution of 0.0833 decimal degrees of latitude and longitude (approximately 5.5 to 8.8 km for the study area comprised between 54 and 18 degrees of latitude north). The statistical methods used in this study required having risk factor variable values for a large set of locations where HPAIV H5N1 or HPAI H5N1 outbreaks would be considered absent (negatives), so as to contrast the agro-ecological conditions associated with HPAIV H5N1 or HPAI H5N1 outbreaks presence (positives). Negatives were selected randomly from throughout the country based on three conditions: i) no HPAI H5N1 outbreaks had been reported and no HPAIV H5N1 positive results had been obtained from the active risk-based surveillance; ii) being at a minimum distance >0.0833 decimal degree of any positive; and iii) being in a location where human population density was >1 person/km^2^ to exclude desert and high mountain areas from the analysis since the focus of this analysis was on locations with likely relevance for disease maintenance in poultry.

### Analysis

Two approaches were used to model the spatial distribution of HPAI H5N1 presence or absence: multiple logistic regression and boosted regression trees (BRT). Logistic regression allows predicting a variable with a binary response, such as the presence or absence of a disease, as a function of a number of variables, or predictors. Logistic regression models have been used in a number of studies trying to identify environmental correlates and risk factors associated with HPAI H5N1 presence [6], [7], [10]. However, one limitation of logistic regression is the necessity to perform specific adjustments to accommodate non-linearity of effect of the continuous-scale risk factors on the logit form of the outcome variable. Two approaches have been described to account for this. First, a risk factor can be added to the model as a quadratic term so that predicted probabilities of presence can be maximum (or minimum) for intermediate values (e.g. [6], [9]). Second, each continuous-scale risk factor variable is converted into a nominal-scale variable where each category level represents a particular range of values in the original variable (e.g. [14], [7], [10]). However, both methods have their limitations. The first method can only partially model more complex non-linear dependencies, and the second method is sensitive to the range of values represented by each category level. In the presence of spatial autocorrelation, logistic regression requires the use of relatively complex estimation algorithms. BRT has been developed relatively recently for predicting the distribution of organisms [15]. It is very efficient for dealing with non-linear relationships and interactions between variables. It can be considered a disadvantage that it does not have the facility to assess the statistical significance of individual effect variables, though it allows estimating the relative importance of each variable to the predictions. In a comprehensive review of presence/absence distribution modeling methods, Elith et al. [16] found BRT to perform best along with the maximum entropy method. Elith et al. [15] published a detailed description of an analysis approach using BRT which implements a cross-validation procedure allowing identification of model parameters. In this study, we compare logistic regression and BRT in terms of validity and ease of interpretation of the outputs. We also discuss our findings in relation to the spatial patterns of HPAI H5N1 described in previously published work that used logistic regression methods.

In the logistic regression method, all variables were forced in the model, and the likelihood ratio test was used to assess the contribution of each variable to the predictions. For the BRT model, we used 10 sets of training and test points for cross-validation, a tree complexity of 4, a learning rate of 0.005 and a bag fraction of 75%. Using those parameters, the cross-validation stepwise function presented by Elith et al. [15] was used to identify the optimal number of trees in the model. The weight of each variable estimated over the identified number of trees was used as an indicator of each variable's importance for predicting HPAI H5N1 presence/absence. One should note that those weights are not absolute metrics, and the weights of all variables of a BRT model sum to 1. This analysis was conducted using two outcome variables, first HPAI H5N1 outbreak occurrence during the entire study period and second HPAIV H5N1 positive findings between 2007 and 2009.

Assessing the performance of our models directly from the logistic regression and BRT predicted probabilities and observed presence/absence had two main limitations. First, logistic regression performance metrics have been shown to be sensitive to low (<10%) and high (>90%) frequencies of the binary outcome categories [17]. The proportion of positives in our dataset was extremely low, and it was therefore necessary to address this potential bias (to our knowledge, the presence of this potential bias has not been thoroughly assessed for BRT models, but see [18]). Second, quantifying model performances using the data set used to train the model tends to inflate the performance metrics compared to a situation where an independent data set is used. We developed a bootstrapping procedure aiming to generate a robust estimate of model performance by simultaneously addressing those two limitations.

The bootstrapping analysis involved a series of sequential steps: (i) selection of a balanced subset of data from the complete dataset: all *n* points with HPAI H5N1 presence were included and an equivalent number of absence points was randomly selected from all ‘absence’ points; (ii) creating a training data set and a test data set: the balanced subset of data was randomly divided into two subsets: one for building the models (training set, with 75% of the points) and the other for evaluating the models (test set, with the remaining 25% of the points); (iii) model development: a logistic regression and a BRT model were built using the training set, and parameters of both models were stored; (iv) model evaluation: the model equations from the logistic regression and BRT models were used to generate predictions using the test set, which in turn were assessed using ROC curves, areas under the curve and Cohen's kappa statistic; (v) risk maps: maps of the predictions produced by each model were stored. Steps (i) to (v) were repeated 50 times, and the mean and standard deviation of all statistics and predicted spatial distributions were estimated.

Due to percentage of post-vaccination seropositivity only being available at province level, using yearly data for the period 2007-2009 as unit of analysis, a separate analysis was conducted to quantify the statistical association between antibody response to vaccination expressed as a percentage and two variables: the presence/absence of HPAI H5N1 outbreak records in the province and detection of H5N1 HPAIV detected through national risk-based surveillance activities conducted by the MoA. The post-vaccination seropositivity was analysed as the response variable of a two-way ANOVA with the presence/absence of HPAI H5N1 outbreak records in the province and the year as two explanatory factors. The same analysis was carried out with the presence/absence of H5N1 HPAIV detected through national risk-based surveillance activities and year as explanatory factors. This allowed separating the effect of HPAI H5N1 status from the possible effect of time.

## Results

**Figure 1:**
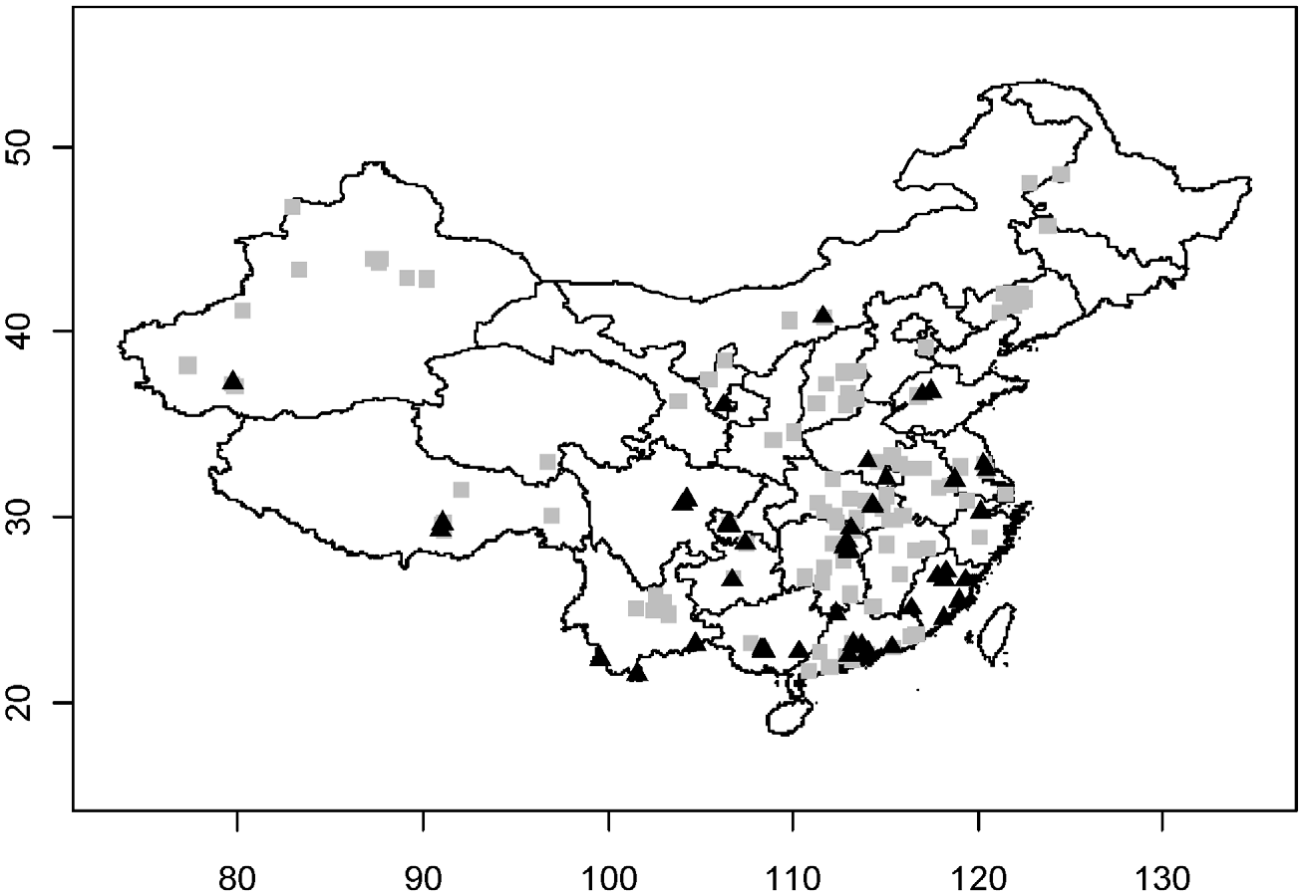
Distribution of HPAI H5N1 outbreaks (grey square) and HPAI H5N1 positive samples identified through surveillance (black triangles) in China.

The distribution of HPAI H5N1 outbreaks and HPAIV H5N1 surveillance positives are shown in Fig. 1. Overall, the two analysis techniques, logistic regression and BRT, provided consistent results in terms of risk factors being identified. In contrast, the set of risk factors and their effect differed strongly between the outcomes of reported HPAI H5N1 outbreak and risk-based surveillance data.

**Figure 2:**
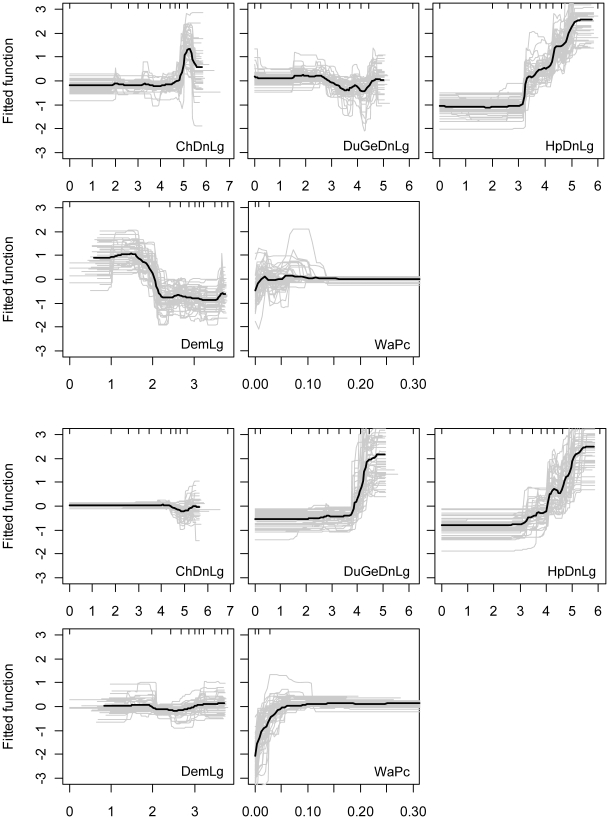
Relationship between risk factors and HPAI H5N1 risk function. The HPAI H5N1 risk functions of the BRT models is plotted as a function of chicken density (ChDnLg, log10 scale), domestic waterfowl density (DuGeDnLg, log10 scale), human population density (HpDnLg, log10 scale), elevation (DemLg, log10 scale), and percentage of land covered by water (WaPc) based on HPAI H5N1 clinical disease outbreak (five first plots) and HPAIV H5N1 risk-based surveillance data (five last plots). The grey lines present the predicted line for each bootstrap, and the black line is the average across all bootstraps.

Based on the logistic regression results, HPAI H5N1 outbreaks were found to be positively associated with human population density and negatively with elevation (Table 2). The BRT models also identified chicken density to be an important variable for discriminating between locations with and without reported HPAI H5N1 outbreaks (BRT weights, Table 2). The averaged BRT model fitted functions shown in Fig. 2 allow a detailed description of these relationships (maps of the predictions coefficient of variation are presented in Figure S1 in Text S1). The predicted risk of HPAI H5N1 outbreak occurrence appears to be constant for densities of chickens ranging from 0 to 10,000 heads/km^2^, then increases to a maximum risk at around 100,000 heads/km^2^. The predicted risk also increases significantly with human population density, starting from a density of 1,000 people/km^2^. We also identify a strong negative relationship with elevation, with the predicted risk function showing two levels, a high risk for elevation ranging from 0–100 m, and a low risk for higher elevations. The predicted risk is relatively flat for all ranges of domestic waterfowl density and percentage of land with surface water.

**Table 2:** Results of the bootstrapped logistic regression model and boosted regression trees applied to clinical HPAI H5N1 disease outbreak and HPAIV H5N1 serological surveillance data.

	Logistic regression				Boosted regression tree	
Variable	Coef (mean)	Coef. (SD)	Ch. Dev. Rem.	p value	Weight (mean)	Weight (SD)
Outbreak data (n pos. = 184)						
Constant	−2.300	1.731				
ChDnLg	0.288	0.340	0.76	n.s.	23.53	9.15
DuGeDnLg	−0.324	0.332	0.80	n.s.	7.22	2.93
HpDnLg	1.544	0.446	31.10	<0.001	28.66	8.54
DemLg	−1.187	0.346	15.46	<0.001	21.08	9.05
WaPc	2.268	7.058	0.85	n.s.	6.71	2.95
RiPc	0.233	1.281	0.42	n.s.	3.26	1.93
Cint	−1.256	0.910	3.41	0.0650	9.54	3.33
Surveillance data (n pos. = 86)						
Constant	−11.404	4.565				
ChDnLg	−1.862	0.835	7.04	0.00797	3.62	2.01
DuGeDnLg	2.120	1.112	10.57	0.00115	36.86	17.21
HpDnLg	2.464	0.701	17.81	<0.001	29.01	15.97
DemLg	0.394	0.813	0.78	n.s.	3.72	2.39
WaPc	20.802	23.022	5.08	0.0242	18.76	12.14
RiPc	−0.571	2.437	0.45	n.s.	2.71	1.57
Cint	−0.434	1.656	0.44	n.s.	5.31	2.22

In contrast, HPAIV H5N1 surveillance positives were found to be positively associated with the density of domestic waterfowl, with percentage of land occupied by water and to human population density (though this factor was not important in the BRT models), and negatively associated with chicken density (Table 2). Here again, the predicted risk function of the BRT models allows a detailed description of these relationships (Fig. 2). The predicted risk of HPAIV H5N1 surveillance positives is constant for waterfowl density ranging between 0 and 10,000 heads/km^2^, then rises sharply for increasing densities. A similar profile as for the HPAI H5N1 outbreak data is found for the association with human population density, with predicted risk increasing with human population density from a density of 1,000 people/km^2^. The predicted risk increases with percentage of area covered by surface water up to a value of approximately 7%, and then remains constant for higher values. The profiles of predicted risk as a function of chicken density and elevation are relatively constant.

**Figure 3:**
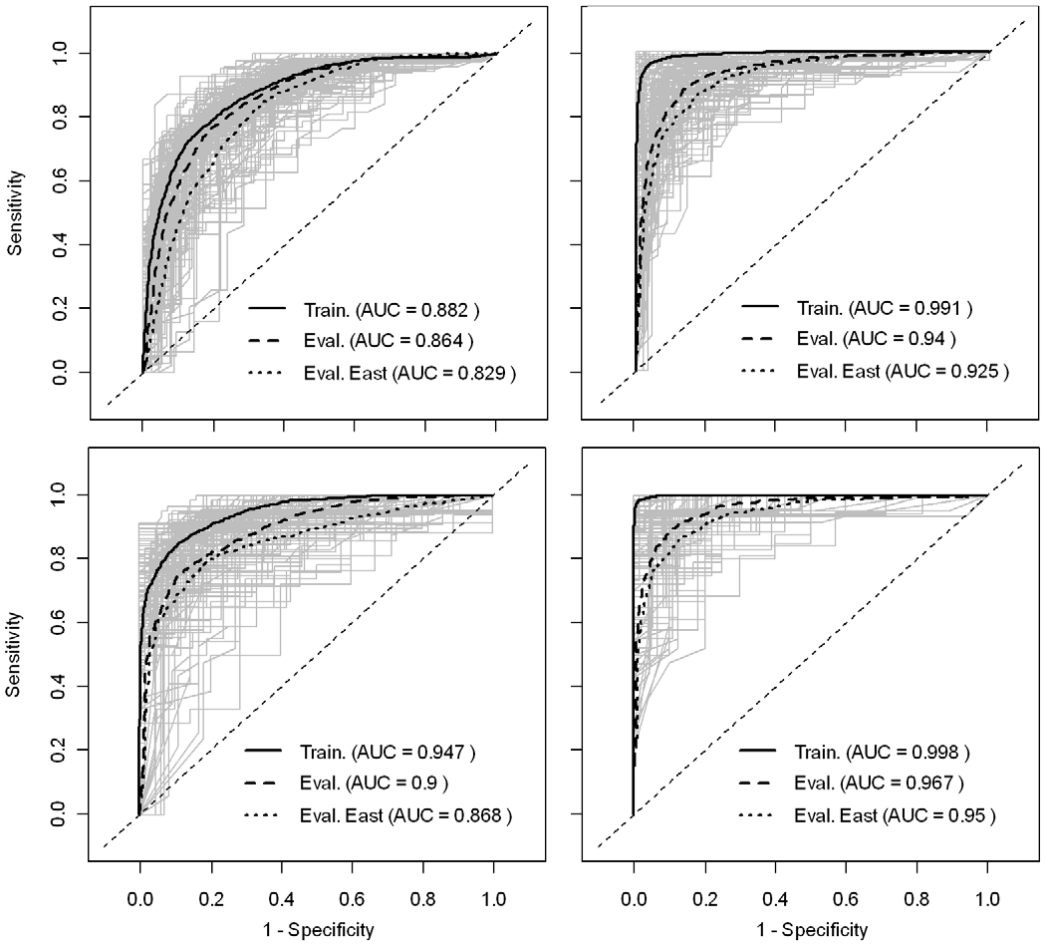
ROC curves of the predicted risk of HPAI H5N1 presence/absence. Left and right plots are the ROC curves of the bootstrapped logistic regression models and BRT models, respectively. Top and bottom plots are the ROC curves of the models based on HPAI H5N1 clinical disease outbreak data, and HPAIV H5N1 risk-based surveillance data, respectively.

The accuracy metrics of the predictions produced by the logistic regression and BRT models are good to excellent, with mean AUC values estimated using the evaluation dataset ranging from 0.864 to 0.967 (Fig. 3). One can note that, as expected, the AUC estimated based on the training data is always better than that estimated using the evaluation dataset, and that this difference is much higher for the BRT models. However, even when assessed using the evaluation dataset, the accuracy of BRT models appears better than that of the logistic regression models. In addition, the accuracy metrics are higher for the models for HPAIV H5N1 risk-based surveillance data than those obtained for the HPAI H5N1 outbreak data. One can note that considering only eastern China in the evaluation of AUC values slightly reduces it's value, but to a marginal extent, showing that the good predictive power does not result from predicting risk over wide desert areas unsuitable to disease spread.

**Figure 4:**
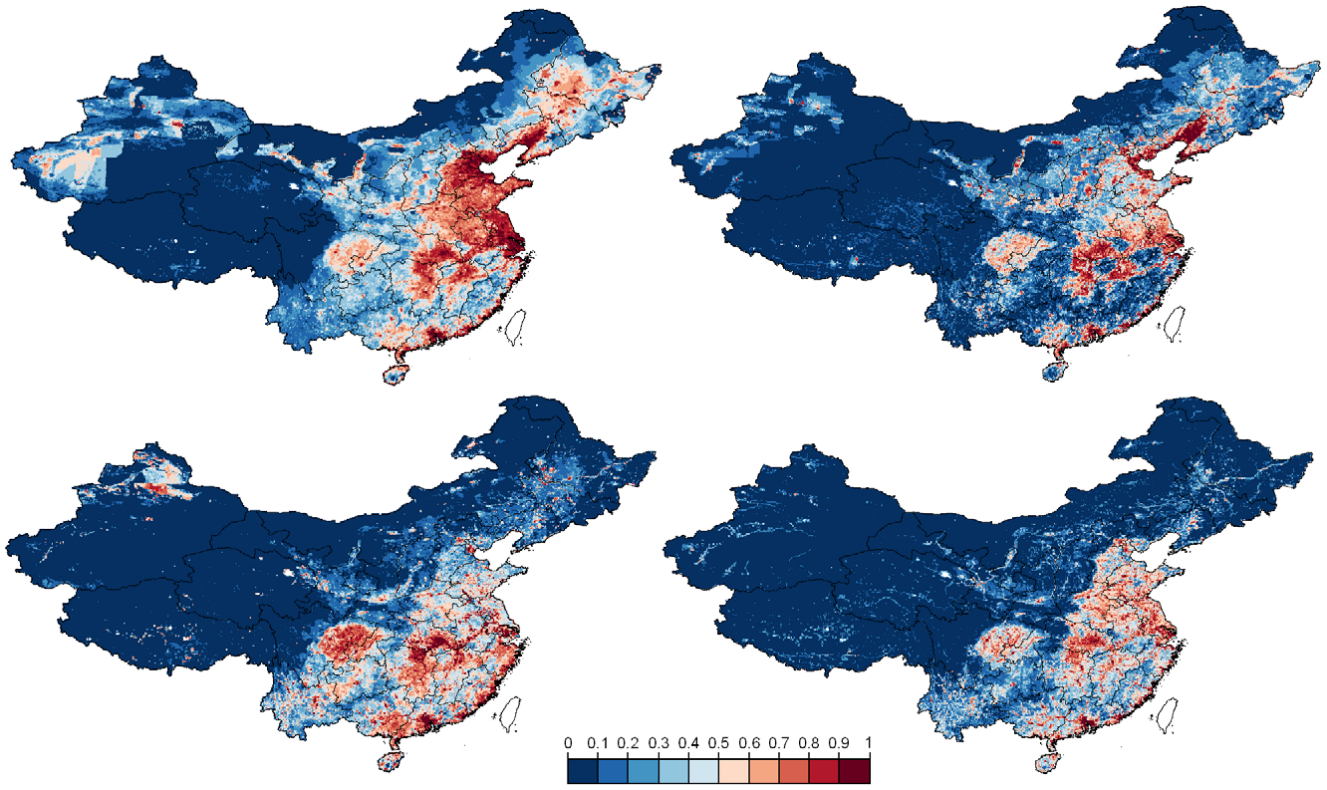
Predicted distribution of HPAI H5N1 risk. Predictions are displayed according to the bootstrapped logistic regression model (left) and boosted regression trees (right), based on reported HPAI H5N1 clinical disease outbreak data (top), or HPAIV H5N1 risk-based surveillance data (bottom).

The predicted geographical distribution of HPAI H5N1 presence also differs according to the type of training data (clinical disease outbreaks vs. risk-based surveillance; Fig. 4). Maps generated based on the outbreak data place more emphasis than those based on risk-based surveillance data on north-eastern regions where chicken densities are higher. We also note a marked difference for the outbreak data between the outputs of the logistic regression model and of the BRT, the latter predicting many more clustered areas with high probability of HPAI H5N1 presence. In contrast, the distribution of predicted HPAIV H5N1 presence based on risk-based surveillance data identifies areas at risk much more concentrated in the southern part of the country, with outputs from the logistic and BRT models showing similar patterns.

**Figure 5:**
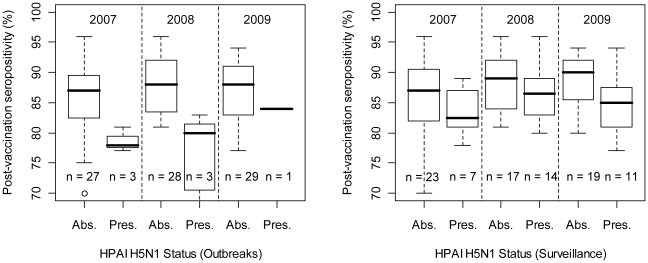
Province-level percentage of post-vaccination sero-positivity as a function of disease/infection status. The left and right plots are based on reported HPAI H5N1 clinical disease outbreak data and HPAIV H5N1 risk-based surveillance data, respectively.

In the analysis at province level, we found that the proportion of seropositivity in post-vaccination surveys was lower in provinces that had reported HPAI H5N1 outbreaks than in those that did not (Fig. 5 left; two-way ANOVA with both year and HPAI H5N1 outbreak status as factor variables; HPAI H5N1 outbreak status: F_1,87_ = 18.53, p<0.001; Year: F_1,87_ = 0.51, n.s.; interaction term - Year by HPAI H5N1 outbreak status: F_1,87_ = 0.0264, n.s.), and in provinces where HPAIV H5N1 had been detected during active surveillance and than in those where this had not been the case (Fig. 5 right; two-way ANOVA with both year and HPAIV H5N1 risk-based surveillance status as factor variables; HPAIV H5N1 risk-based surveillance status: F_1,87_ = 5.09, p = 0.026; Year: F_1,87_ = 2.02, n.s.; interaction term - year by HPAIV H5N1 risk-based surveillance status: F_1,87_ = 0.172, n.s.).

## Discussion

In China, where approximately 15 billion head of poultry are produced annually with a standing population of 5.6 billion chickens, 760 million ducks and 300 million geese, major regional differences are apparent in ecological systems, husbandry practices, cultural behaviors and economic development with a consequential impact on the distribution of infectious diseases including HPAI H5N1, as well as their maintenance and spread and therefore on disease control options.

To date, spatial studies aiming at identifying HPAI H5N1 risk factors have been undertaken in many countries where the disease was introduced such as Thailand and Vietnam [5], [6], [14], [8], [7], [19], Korea [20], India and Bangladesh [9], [11], [21], Romania [22], [23] or Africa [24], [25]. Only three studies analyzed the distribution of HPAI H5N1 outbreaks in China [4], [26]. Of these, only the study by Fang et al. [4] attempts to map the distribution of HPAI H5N1 risk. Whilst highly valuable given that it is the first analysis, the output predicts areas at high risk in ecological areas that would not support the maintenance and transmission of the virus such as in the extremely large desert regions of Inner Mongolia, Tibet and Xinjiang autonomous regions.

In our study, we reported different results for the analyses based on outbreak and risk-based surveillance data. The distribution of reported HPAI H5N1 outbreaks was found to be primarily associated with lowland regions with high human population and chicken density. In contrast, HPAIV H5N1 presence detected through risk-based surveillance activities was found to be associated with regions with high waterfowl densities and were covered by high proportions of surface water. This result is very interesting since it may be a reflection of differences in HPAIV H5N1 pathogenicity between chickens and ducks, combined with environmental and host population conditions supporting virus spread and clinical disease outbreak occurrence as distinct from clinically silent virus persistence.

HPAI H5N1 is far more pathogenic in chickens than in ducks [27], [28], though there is also evidence of significant variability in virulence at the species level [29]. In the absence of control or prevention measures, the spread of HPAIV H5N1 and occurrence of clinical disease outbreaks is facilitated in regions where the density of chickens is particularly high, especially in intensive and industrial conditions where high numbers of animals are together facilitating transmission. Such regions are encountered in the north-eastern part of China, where the low cost of grain feed production and a fast-rising demand for poultry meat has supported the rapid development of intensive chicken production. Those intensive poultry production systems invest significant resources in disease prevention measures, and will apply mass-vaccination of their flocks, thereby preventing HPAIV H5N1 spread within and between farms. However, it is likely given the exceptionally high density of chickens and farms that occasional, albeit rare, lapses in vaccination coverage result in a small number of outbreaks. The human population density risk factor can be interpreted as a proxy of several epidemiological processes that are more likely to occur in highly-populated areas, such as a higher likelihood of outbreak detection and higher possibilities of HPAIV H5N1 transmission through trade and farming-related activities.

In contrast, long-term persistence of HPAIV H5N1 can only be possible if the virus can circulate without being detected or reported. Domestic ducks have been shown to be able to excrete large amounts of virus whilst remaining apparently healthy [27]. Regions rich in domestic waterfowl are hence more prone to long-term persistence of HPAIV H5N1. This can be further exacerbated in geographical areas with an abundance of surface water. Permanent water bodies, rivers, rice paddy fields and canals are the habitat of wild and domestic ducks. One may speculate that water facilitates the transmission between hosts without direct contact through the faecal excretion of the virus, its persistence in the water, and the oral infection of other susceptible hosts sharing the same pond or downstream canal or river. The results indicating that HPAIV H5N1 presence detected through risk-based surveillance is associated with areas that have high waterfowl densities and a high proportion of surface water allows thus a straightforward interpretation. Associations between HPAIV H5N1 and domestic duck density had already been identified in other countries [5], [7]. However, no difference between outbreaks and clinically-silent infections was made in these earlier studies, which indeed becomes essential when analyzing HPAIV H5N1 distribution in the context of mass-vaccination such as in China.

Interestingly, the farming and cultural practices encountered in these regions were already described by Shortridge 28 years ago as an avian influenza breeding ground [30]. Among others, Southern China still hosts a massive duck population raised on ponds and rice fields, facilitating frequent faecal-oral transmission of multiple influenza subtypes leading to a year-round and inter-epidemic occurrence of influenza viruses. Historically, agricultural practices in China have developed from the need to feed the people as efficiently as possible, using all available resources, and with little recourse to modern farming methods. Domestic ducks were first moved from rivers to cultivated rice fields at the start of the Qing dynasty in the middle of the 17th century [31], [32] to help protect the growing rice from pests. This practice reduces farmers' dependence on chemical insecticides, herbicides, fertilizers and mechanical farming aids and provides a close association between bird, water, rice and people. Ducks raised in ponds are also an important feature in the villages and communities of China, especially in southern China and coastal areas including the waterways of the Pearl River delta which are ideal for rice and fish farming [30]. Furthermore, southern China has always been the focus of influenza experts' attention and often been referred as a hypothetical epicenter of AI pandemic strains. The foundation of this concept was originally raised by Webster et al. [33] and supported by the wide variety of influenza virus subtypes discovered in Southern China during decades [34]–[37]. More specifically, the distribution of HPAIV H5N1 risk of persistence inferred from the risk-based virological surveillance data and using the logistic and BRT models is similar and highlights different levels of risk according to the following ecological regions (Fig. 4, bottom; see Figure S2 in Text S1 for a map of the zones):

Zone I) In Southern China a large potential zone of virus persistence extends from south of the Yangtze River. This area hosts the vast majority of the Chinese duck population comprising birds for meat or egg production. This area can be subdivided into three areas: I-a) an area which extends from the provinces of Jiangsu, Anhui, Hubei, Jiangxi, Hunan, Guangxi autonomous region down to Guangdong province. This might be one of the most important ecological zones where key epidemiological drivers for emergence, persistence and spread are present, including a huge reservoir population, traditional farming system, a high animal and human population density, some major wild bird congregation sites such as the Poyang lake located in Jiangxi province and an important North – South gradient of poultry trade which crosses this region. This supports the hypothesis of a wider and slightly displaced epicenter of influenza viruses, not only concentrated around the Pearl River delta in Guangdong province but extending south of the Yangtze River and including provinces such as Jiangxi where internal segments of the 1996 geese HPAI H5N1 virus may have originated [38]. I-b) A coastal area stretching from Jiangsu to Guangdong provinces with a risk hotspot in Guangdong province along the Pearl River delta. This strip of coastal land also hosts the typical duck pond system where the risk of infection and disease is present. I-c) Few isolated areas within this geographical zone displaying an increased risk located in Yunnan, Guangxi autonomous region, Guizhou, Sichuan and Chongqing provinces which have experienced either outbreaks in the past (Guangxi autonomous region and Yunnan) or only reported viral circulation (Sichuan and Chongqing provinces).

Zone II) A vast geographical area in the West and North, displaying radically different geography, socio-economic and animal production features and characterized by scattered and isolated spots of higher predicted risk. This includes specifically southern Tibet autonomous region and scattered areas in the North and South of Xinjiang autonomous region where sporadic outbreaks have occurred in the past.

Zone III) In the North-East of the Yangtze River, a region where the contribution to disease persistence seems fairly limited while localized areas at higher risk of outbreaks encroach regions of intensive production where the disease could rapidly spread in case of virus introduction and breach in biosecurity. This region extends from Shangdong into Liaoning, Jilin and Helongjiang provinces. These provinces are characterized by denser human population and large-scale commercial poultry production, and were predicted as high risk based on the reported clinical disease outbreak data (Fig. 4 top). In these regions of North-Eastern China, chicken production and marketing systems are intensifying and concentrating in response to economic growth and urbanization. Substantial numbers of poultry are now processed at large-scale slaughterhouses in this region, while the majority of poultry are still sold through live poultry markets in the South of the country. In the colder north-eastern provinces water birds are also housed and kept more intensively.

The apparent persistence of HPAIV H5N1 in those regions has two main implications. First, given the possible presence of silent infection involving an extremely high population of domestic waterfowl, eradication of the virus through massive vaccination appears extremely difficult, although it has successfully reduced the number of outbreaks. Vaccination has been one major component of the government policy to curb the spread of the disease and reduce the incidence of outbreaks of clinical disease and of transmission of infection. China uses more vaccine against avian influenza than any other country and Chinese veterinary authorities base much of their control and preventive strategy around vaccination [39]–[41]. More than 13 billion doses of AI vaccine have been used each year since 2007 [39] and the objectives of the national strategy are to reach a 100% vaccination coverage for the national poultry population and to ensure an effective immune response (defined as sero-conversion in bird with titres >4Log2 when measured by HI test) in more than 70% of the nationwide poultry population all year round. In this study, we also analyzed the post-vaccination surveillance data collected at provincial level since January 2007 and found that provinces where clinical HPAI H5N1 outbreaks had been reported or HPAIV H5N1 detected had a lower level of post-vaccination seropositivity, confirming that increased protection does indeed result in lower disease outbreak or infection risks but would require an approach better targeted at identified high risk areas to drastically reduce the viral load in the environment.

Second, the different regions of China are not independent and are possibly epidemiologically linked through poultry trade and likely also through wild bird migrations. High production-demand discrepancies lead to long-distance trade of poultry products (e.g. chicken from the north exported to southern provinces, or duck meat exported from the south to the north). In addition, areas such as the Poyang lake, where a large population of domestic waterfowl is raised in close proximity to thousands of over-wintering wild waterfowl, could favour the transmission between wild and domestic waterfowl and lead to long-distance transmission of the virus. As a consequence, the persistence of HPAIV H5N1 in some particular regions may influence the chances of introduction into other more distant regions. For instance, the wild bird 2.2 clade which was associated with the origin of the Qinghai lake epidemic in 2005 in West China was responsible for a major outbreak during the same year in domestic poultry in Liaoning province, in the north eastern part of the country. Likewise, the 2006 Shanxi strain also grouped into the clade 7 cluster present in North-Central China has been found in Jiangsu province in South Eastern China. There is a complex pattern of links that exists between these different ecological regions that offers hiatuses for viruses to escape their reservoir areas and invade others.

Continued efforts pursued by the Ministry of Agriculture, its affiliated research centers and local veterinary authorities to strengthen the HPAI national surveillance program and its control strategy have resulted in a steady decrease in the number of outbreaks reported since 2004 and a better understanding of HPAIV H5N1 infection distribution in space, time and within traditional marketing systems known as live bird markets.

However, national surveillance programs have also demonstrated that HPAIV H5N1 continues to circulate in poultry on a regular basis. Since 2007, an apparent increase in virus detection is believed to represent the result of increased and intensive efforts made by the Ministry of Agriculture to detect the virus through targeted risk-based surveillance activities at live bird markets and high-risk farms in a context of massive vaccination efforts that could potentially mask the clinical expression of the disease within a large population of immunized birds.

Although the epidemiology of HPAIV H5N1 in China does not seem to present radically different features compared with neighboring countries also affected by the disease, it remains unique in terms of the abundance of reservoir species both domestic or wild, providing ample opportunities for a sustained and rapid evolution of the virus and requiring intensive virus monitoring for pandemic preparedness matters. While revisiting the concept of epicenter for pandemic strains of avian origin, the results of this study represent major improvements over previous efforts in mapping the risk of HPAI H5N1 in two main aspects.

First, it allows identifying several risk factors of animal, environmental and anthropogenic nature, with clear biological and epidemiological interpretation. Second, the bootstrapped statistical modeling allows us to robustly estimate the predictive power of our model, but also to map the uncertainty that goes with our predictions (Figure S1 in Text S1), which is useful information for an applied use of these maps.

Combining innovative modeling techniques with data of improved quality and integrating measures of infection persistence, our results have broad fundamental implications in a country where understanding of the ecology of influenza viruses, although of utmost importance for pandemic preparedness purposes, has remained until now mostly speculative.

Finally, the analyses presented in this study may be improved in the future by several complementary approaches. First, the potential transmission through trade patterns and bird migration should be more comprehensively assessed. An increasing amount of data are being collected on both aspects, and this will ultimately contribute to better understanding of how areas of high potential for HPAIV H5N1 persistence may be connected to each others. Second, the results could be further integrated into an Asia-wide improved understanding of HPAIV H5N1 distribution models, benefiting from several studies that have been undertaken in neighboring countries. Third, information on true negatives obtained through the national surveillance programme would reduce the risk of including false-negatives in the analyses, and provide higher resolution estimates of the relative importance of risk factors.

## Supporting Information

Text S1 Supplementary information Figure S1 and Figure S2.
(0.61 MB DOC)
