# Supplementary material for: Spatial Distribution and Risk Factors of Highly Pathogenic Avian Influenza (HPAI) H5N1 in China
Source: PLoS Pathog. 2011 Mar 3;7(3):e1001308. doi: 10.1371/journal.ppat.1001308 (PMC3048366; doi:10.1371/journal.ppat.1001308)

Text S1

Figure S1 Coefficient of variation of the predicted distribution of HPAI H5N1 risk according to the bootstrapped logistic regression model (left) and boosted regression trees (right), based on reported HPAI H5N1 clinical disease outbreak data (top), or HPAIV H5N1 risk-based surveillance data (bottom).


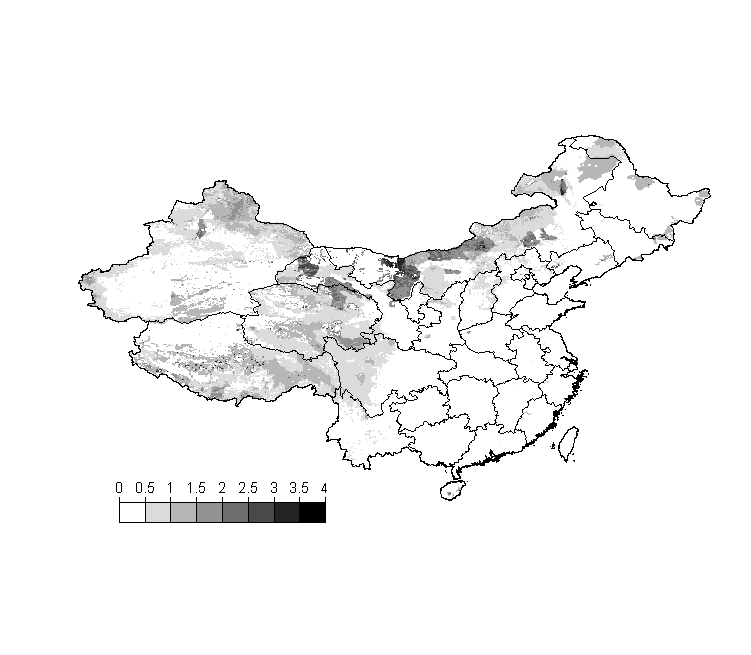

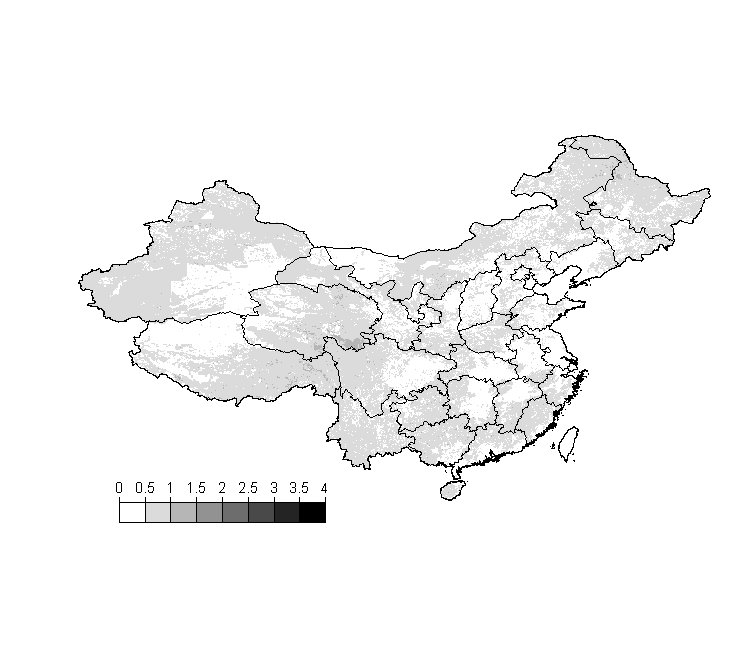


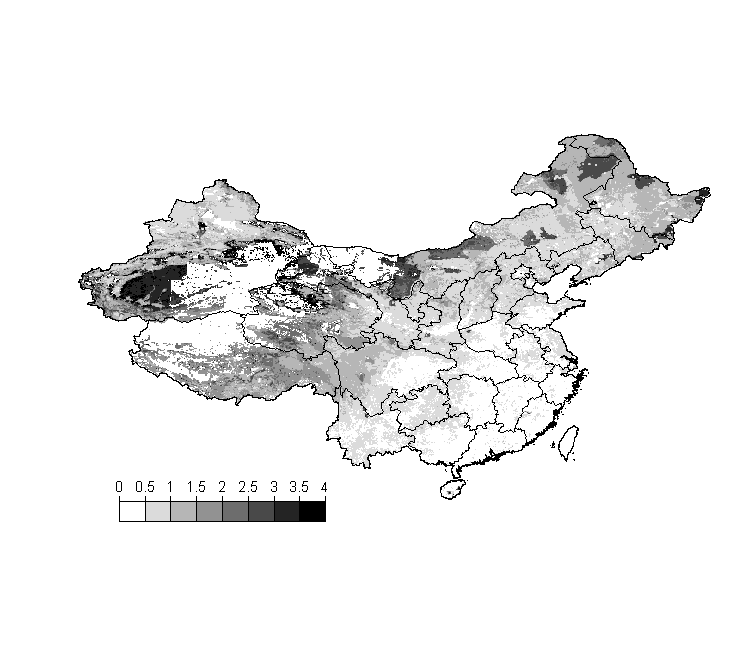

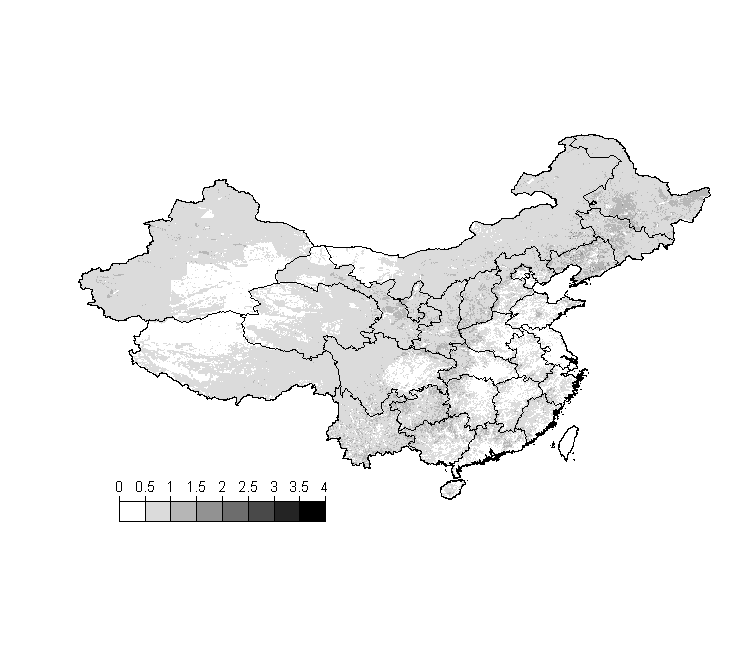


Figure S2 Map of the different epidemiological zones discussed in the paper (the distinction between these zones is provided for illustrative purpose only)


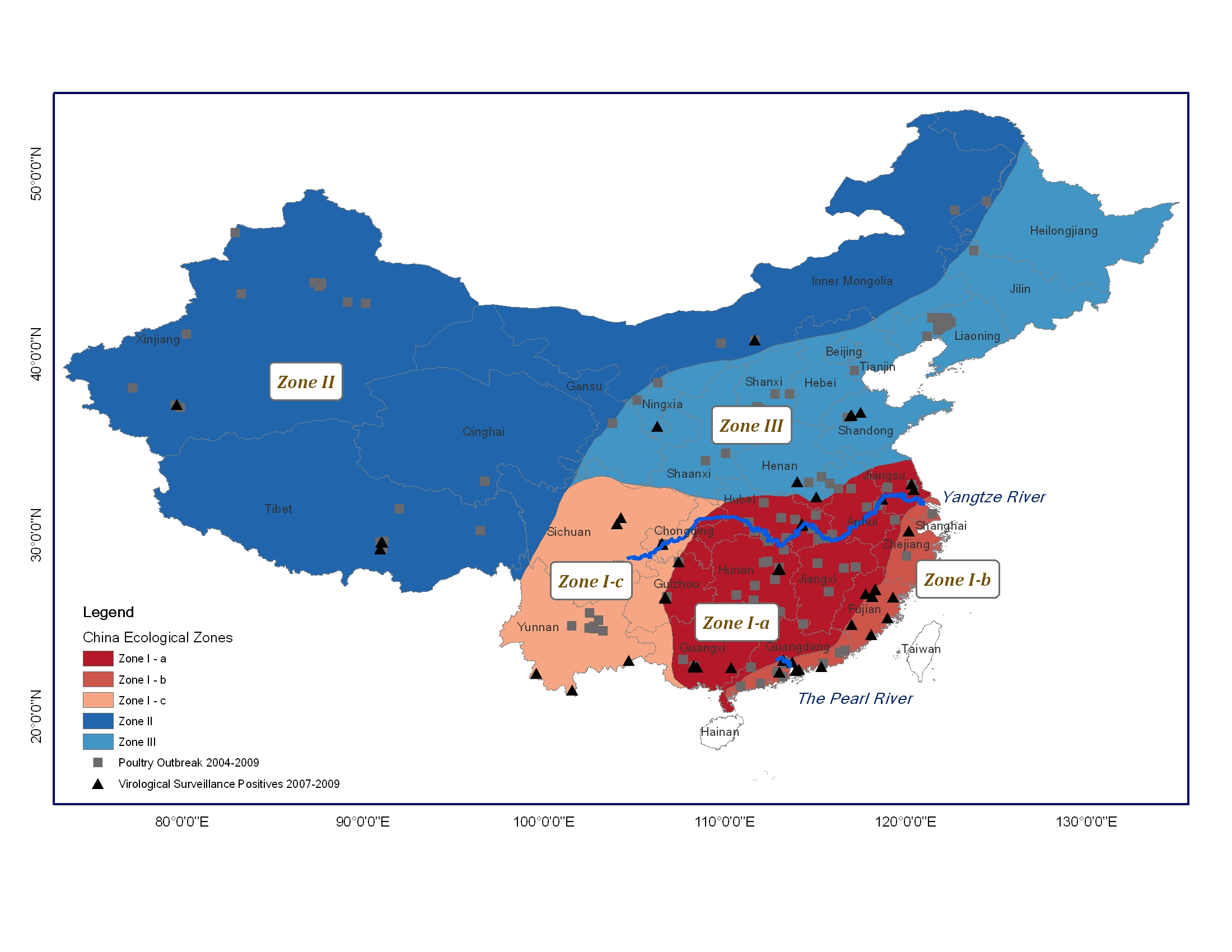

Supplement: Text S1 — Supplementary information Figure S1 and Figure S2. (0.61 MB DOC) [file ppat.1001308.s001.doc]
